# Conserved Immune Recognition Hierarchy of Mycobacterial PE/PPE Proteins during Infection in Natural Hosts

**DOI:** 10.1371/journal.pone.0040890

**Published:** 2012-08-01

**Authors:** H. Martin Vordermeier, R. Glyn Hewinson, Robert J. Wilkinson, Katalin A. Wilkinson, Hannah P. Gideon, Douglas B. Young, Samantha L. Sampson

**Affiliations:** 1 TB Research Group, Animal Health and Veterinary Laboratories Agency-Weybridge, New Haw, Addlestone, United Kingdom; 2 Clinical Infectious Diseases Research Initiative, Institute of Infectious Disease and Molecular Medicine and Department of Medicine, Faculty of Health Sciences, University of Cape Town, Cape Town, South Africa; 3 Division of Mycobacterial Research, MRC National Institute for Medical Research, London, United Kingdom; 4 Department of Medicine, Imperial College London, London, United Kingdom; Universita di Sassari, Italy

## Abstract

The *Mycobacterium tuberculosis* genome contains two large gene families encoding proteins of unknown function, characterized by conserved N-terminal proline and glutamate (PE and PPE) motifs. The presence of a large number of PE/PPE proteins with repetitive domains and evidence of strain variation has given rise to the suggestion that these proteins may play a role in immune evasion via antigenic variation, while emerging data suggests that some family members may play important roles in mycobacterial pathogenesis. In this study, we examined cellular immune responses to a panel of 36 PE/PPE proteins during human and bovine infection. We observed a distinct hierarchy of immune recognition, reflected both in the repertoire of PE/PPE peptide recognition in individual cows and humans and in the magnitude of IFN-γ responses elicited by stimulation of sensitized host cells. The pattern of immunodominance was strikingly similar between cattle that had been experimentally infected with *Mycobacterium bovis* and humans naturally infected with clinical isolates of *M. tuberculosis.* The same pattern was maintained as disease progressed throughout a four-month course of infection in cattle, and between humans with latent as well as active tuberculosis. Detailed analysis of PE/PPE responses at the peptide level suggests that antigenic cross-reactivity amongst related family members is a major determinant in the observed differences in immune hierarchy. Taken together, these results demonstrate that a subset of PE/PPE proteins are major targets of the cellular immune response to tuberculosis, and are recognized at multiple stages of infection and in different disease states. Thus this work identifies a number of novel antigens that could find application in vaccine development, and provides new insights into PE/PPE biology.

## Introduction

The global public health and economic impact of infection with *Mycobacterium tuberculosis* and the closely related *Mycobacterium bovis* is severe. Approximately 9.8 million new cases of *M. tuberculosis* infections were estimated for 2010 [1], and recent years have seen a rapid increase in *M. bovis* infections in the UK [2]. To curb the spread of infection, improved therapeutic, diagnostic and preventative tools are urgently required. Such developments will be underpinned by an improved understanding of pathogen biology and host immunology.

A number of mycobacterial molecules have been implicated in TB pathogenesis and shown to modulate host responses to infection. These include members of the intriguing PE/PPE protein families that are named for conserved proline-glutamate (PE) and proline-proline-glutamate (PPE) motifs near their N-termini [3]. These large protein families are especially abundant in pathogenic mycobacteria, with almost 200 *pe/ppe* genes in both *M. tuberculosis* and *M. bovis* [3], [4]. The biological role of these proteins remains elusive. Sequence homology-based inferences have shed little light on their function, and structure-based predictions are limited by the availability of only one experimentally determined PE/PPE structure [5]. High-throughput phenotype screening approaches have demonstrated that very few family members are essential for *in vitro* growth or *in vivo* survival [6], [7], [8], which could be indicative of functional redundancy. Speculation regarding the function of these proteins abounds, and the identification of the multi-member family prompted the suggestion that PE/PPE proteins may act as variable antigens [3]. “Antigenic variation” usually refers to the phenomenon of regulated switching between functionally conserved, but immunologically distinct, members of a multigene family, which can aid in the avoidance of antigen-specific immune selection [9]. In particular, the highly polymorphic PE-PGRS (polymorphic GC-rich sequence) and PPE-MPTR (major polymorphic tandem repeat) are speculated to aid in immune avoidance in this way. Although this hypothesis has yet to be experimentally verified, there are indications that PE/PPE proteins may contribute to evasion of host immunity by other non-mutually exclusive mechanisms [10]. For example, Ramakrishnan and colleagues elegantly demonstrated that a *Mycobacterium marinum pe-pgrs* gene is required for granuloma persistence and survival in macrophages [11]. As their name suggests, the PE-PGRS subgroup of the PE family incorporates highly repetitive, variable C-terminal domains, and it has been shown that these domains can inhibit processing and subsequent antigenic presentation [12], [13]. Others have demonstrated that selected PE/PPE family members are involved in modulating cytokine secretion and other responses (such as apoptosis and DC maturation) via interaction with toll-like receptor 2 (TLR-2) [14], [15], [16], [17]. Recently, deletion of a *pe-pgrs* family member was shown to attenuate *M. tuberculosis* [18]. Available data therefore suggests that different PE/PPE proteins may play distinct roles during infection, which are not yet fully understood.

Some functional clues may be provided by the close genomic, evolutionary and functional links between the *pe/ppe* genes and the *esx* gene clusters [19]. The *M. tuberculosis* genome contains 5 *esx* gene clusters, including the *esx-1* locus, which encodes the ESX-1 secretion system. ESX-1, along with its best-studied substrates, EsxA (ESAT-6, early secreted antigenic target of 6 kDa) and EsxB (CFP-10, culture filtrate protein of 10 kDa), has been implicated in mycobacterial virulence [20], [21], [22]. A subset of the PE/PPE protein family is encoded as co-transcribed *pe/ppe* gene pairs, embedded within the *esx-1, -2, -3* and *-5* regions, immediately upstream of an *esxB/esxA* paralogous pair ([19], Figure S1). These will subsequently be referred to as *pe/ppe* genes from complete *esx* clusters. Another 5 *pe/ppe* gene pairs are found immediately adjacent to an *esxB/esxA* gene pair with no other *esx-*region genes ([19], Figure S1); these will be referred to *pe/ppe* genes from partial *esx* clusters. Finally, there are 8 examples of isolated *pe/ppe* gene pairs, with no adjacent *esx* genes ([19], Figure S1). Multi-genome comparisons and phylogenetic analysis suggests that *pe/ppe* evolution and expansion is closely linked with that of the *esx* gene clusters ([19]). Furthermore, an apparent functional link between the PE/PPE families and the ESX secretion systems has been elucidated; a series of studies suggests that the secretion of a large number of PE/PPE proteins in *M. marinum* is ESX-5-dependent [23], [24], [25]. More recently, it has been demonstrated that the *M. tuberculosis* ESX-5 region is required for the secretion of PPE41 [26]. Although it is not yet known whether their localization is always ESX-mediated, cell wall association and/or secretion have been demonstrated for several PE/PPE family members [10]. Importantly, cell surface exposure and secretion into the extracellular milieu places these proteins in an ideal position to interact with host cell components and in particular to evoke T cell responses.

Selected members of these families have been shown to elicit robust immune responses in infected hosts. PE/PPE proteins elicit cellular and/or humoral responses [10], leading to suggestions that they be further evaluated as possible vaccine and diagnostic candidates. Indeed, the subunit vaccine candidate Mtb72F, currently undergoing clinical trials, is based on a chimeric fusion of a PPE (Rv1196/PPE18) protein with a protease [27], [28], [29], [30]. However, studies to date have tended to investigate only a limited number of PE/PPE proteins, and this lack of a systematic approach limits our overall understanding of the immunogenic potential of these proteins. To address this shortcoming, we performed comprehensive analysis of the immunogenicity of 36 PE/PPE proteins in cattle infected with *M. bovis* and in *M. tuberculosis*-infected humans, both of which represent infections in their natural hosts. We have identified a large number of immunogenic PE/PPE-derived peptides, and show that antigenic cross-reactivity is associated with immune recognition of PE/PPE proteins. Our results demonstrate a similar immune recognition hierarchy in humans and cattle, and provide insight into the biology of these intriguing proteins.

## Results

### PE/PPE Protein Selection

A total of 36 PE/PPE proteins were selected for this study: 10 of these were encoded by *pe/ppe* genes within complete *esx* regions (*esx-1,-2,-3 and -5*); 10 were encoded by *pe/ppe* genes from partial *esx* clusters, adjacent to *esxB/esxA* homologues (but with no other adjacent *esx* region genes), and 16 were encoded by isolated *pe/ppe* pairs (Table 1, Figure S1). We had previously demonstrated that this subset of PE/PPE proteins encoded within *esx*, partial *esx* and non-*esx* genomic locations represented 4 phylogenetic sub-groups ([19], Figure S2). The rationale behind this selection was to allow analysis of a diverse cross-section of the PE/PPE family.

**Table 1:** PE/PPE proteins selected for this study.

Rv number	PE/PPE	ESX association^a^	# peptides (# pools)	*M. bovis* sequence comparison
Rv0285	PE5	C	12 (1)	Identical
Rv0286	PPE4	C	63 (6)	Identical
Rv0915c	PPE14	I	52 (5)	Identical
Rv0916c	PE7	I	11 (1)	Identical
Rv1039c	PPE15	P	48 (5)	Identical
Rv1040c	PE8	P	33 (3)	Identical
Rv1168c	PPE17	I	42 (4)	Frameshift in *M. bovis*, otherwise identical
Rv1169c	PE11	I	11 (1)	Identical
Rv1195	PE13	P	11 (1^b^)	Identical
Rv1196	PPE18	P	48 (5)	R235L, M249V, A275del, Q279E
Rv1386	PE15	I	12 (1)	Identical
Rv1387	PPE20	I	66 (6)	V94A
Rv1787	PPE25	C	45 (4)	S309del,V311G
Rv1788	PE18	C	11 (1)	Identical
Rv1789	PPE26	C	48 (5)	S274A
Rv1790	PPE27	C	10^c^ (1)	P190S
Rv1806	PE20	I	11 (1)	Identical
Rv1807	PPE31	I	48 (5)	F223S, V234L
Rv2107	PE22	I	11 (1)	Identical
Rv2108	PPE36	I	29 (3)	Identical
Rv2430c	PPE41	I	23 (2)	Identical
Rv2431c	PE25	I	11 (1)	Identical
Rv2768c	PPE43	I	47 (5)	P263R
Rv2769c	PE27	I	33 (3)	P54S, V270M
Rv3018A	PE27A	P	1 (1^b^)	Identical
Rv3018c	PPE46	P	19^c^ (2)	Identical
Rv3021c	PPE47	P	44 (1)	G222A, L240V, A242T
Rv3022A	PE29	P	11^c^ (1)	Identical
Rv3477	PE31	I	11 (1)	Identical
Rv3478	PPE60	I	48^c^ (5)	Identical
Rv3621c	PPE65	P	51 (5)	Deleted in *M. bovis*
Rv3622c	PE32	P	11 (1)	Deleted in *M. bovis*
Rv3872	PE35	C	11 (1)	Identical
Rv3873	PPE68	C	45 (4)	Identical
Rv3892c	PPE69	C	49 (5)	T19K
Rv3893c	PE36	C	9 (1)	Identical

**a.** C, PE/PPEs encoded by complete *esx* gene cluster; P, PE/PPE encoded by partial *esx* cluster; I, PE/PPE encoded by isolated *pe/ppe* pair (see Figure S1).

**b.** Single peptide for Rv3018A/PE27A mixed with PE13 set.

**c.** Pool does not include all possible overlapping peptides, some were excluded due to 100% identity.

Peptides (n = 1043) were designed based on the *M. tuberculosis* H37Rv protein sequences, and with the exception of one PE/PPE pair, all of the proteins selected for this study were present in *Mycobacterium bovis* (Table 1). The protein pair PE32/PPE65 is encoded within a 5894 bp region in *M. tuberculosis* H37Rv which is deleted in *M. bovis*, thus these orthologues are not present in *M. bovis.* Twenty-three proteins selected for the study were 100% identical in the two species (Table 1). Mutations identified in the remaining 11 proteins are summarized in Table 1. In total, these differences affected only 30 peptides in 12 pools. (Table S1). We therefore concluded that the peptide library based on *M. tuberculosis* H37Rv was suitable for use in both species.

### PE/PPE Immune Recognition in *M. bovis*-infected Cattle

**Figure 1:**
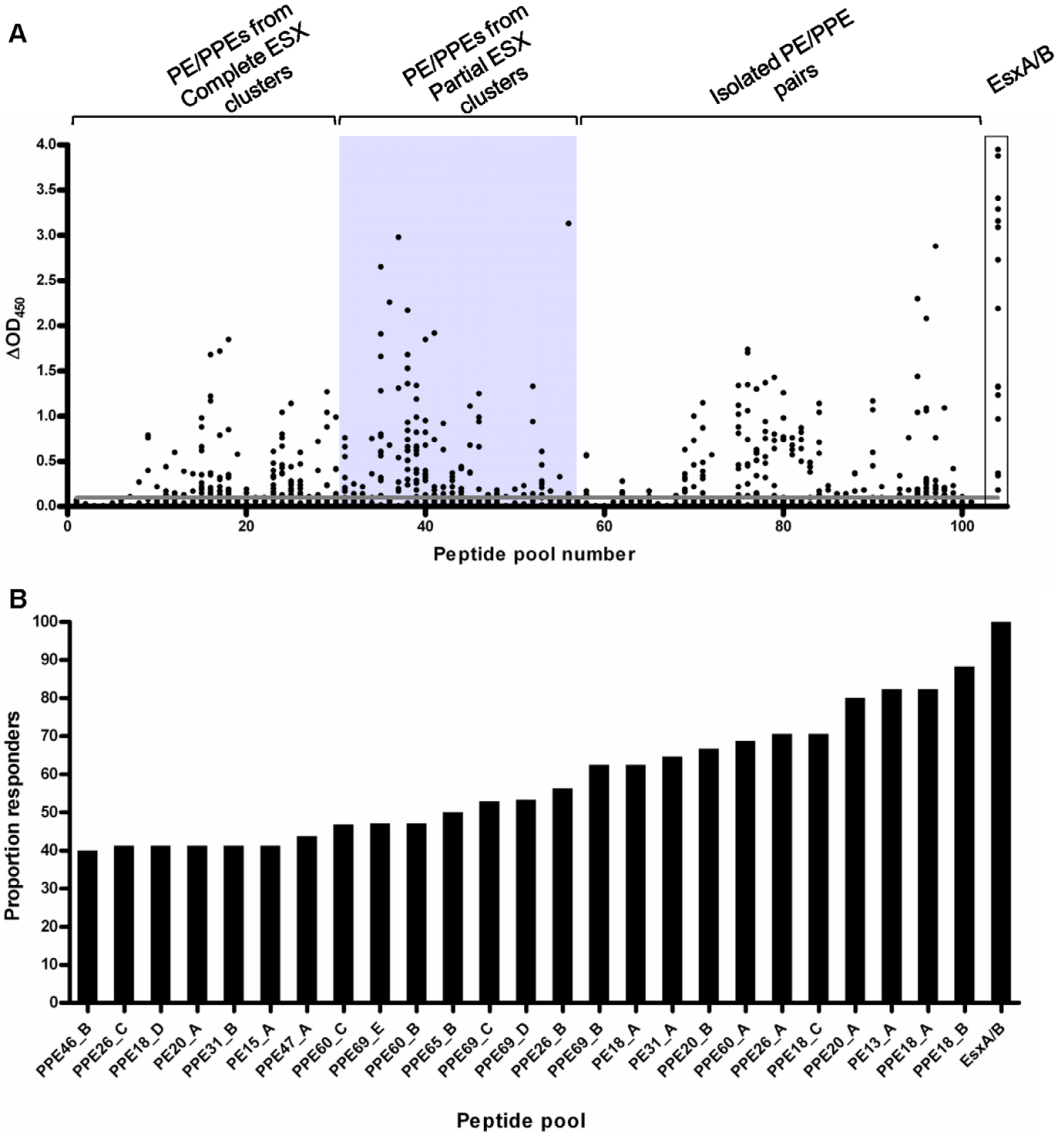
IFN-γ responses to PE/PPE peptide pools in experimentally infected cattle. Whole blood from cattle experimentally infected with 3×10^3^ CFU *M. bovis* AF2122/97 was stimulated with pools of peptides representing 36 PE/PPE proteins at 12–14 weeks post-infection. IFN-γ production was assessed by the ELISA-based Bovigam assay. (A) IFN-γ responses to 101 PE/PPE peptide pools in experimentally infected cattle (n = 17) expressed as OD_450_– Nil antigen well reading (ΔOD_450_). Grey box highlights responses to PE/PPE peptides derived from PE/PPE proteins encoded by partial *esx* regions. Peptides derived from PE/PPE proteins from complete ESX regions and those encoded as isolates PE/PPE pairs are also indicated. Responses to an EsxA/B peptide cocktail are also shown for comparison. (B) Twenty-five PE/PPE pools that elicited positive responses in ≥40% cattle tested, ranked according to proportion responders.

To gain an overview of the degree of immunogenicity of the PE/PPE proteins selected for this study, we first performed a cross-sectional study in cattle experimentally infected with *M. bovis.* Samples were obtained from 17 animals at 12–14 weeks post-infection, and recall immune responses to peptide pools corresponding to 36 PE/PPE proteins were analyzed. Whole blood was stimulated with 101 pools of 8–12 peptides per pool (n = 1043 total peptides), and IFN-γ production was measured by ELISA. This approach identified a striking number of immunogenic PE/PPE peptide pools (Figure 1A). Sixty-five peptide pools elicited a positive response in 2 or more animals. Fifteen pools, representing 9 different PE/PPE proteins, were recognized in ≥50% of experimentally infected cattle (Figure 1B, Table 2, Table S1). Ten animals (58.8%) recognized ≥20 pools each (mean number of pools recognized, 23.1; SD, 16.2; range, 57). Notably, in 9 skin-test and Bovigam-negative animals tested prior to experimental infection, no response to any of the PE/PPE peptide pools was detected (data not shown), indicating the specificity of these immune responses.

**Table 2:** Bovine and human responses to selected PE/PPE peptide pools.

	Cattle		Humans	
*Pool*	*% responders^a^*	*Mean* *ΔOD_450_*	*% responders^a^*	*Median* *SFC*
PPE18_B	88.2	0.52	58.8	28
PE13_A	82.4	0.72	52.9	20
PPE18_A	82.4	0.72	70.6	24
PPE20 A	80.0	0.41	64.7	28
PPE18_C	70.6	0.39	29.4	8
PPE26_A	70.6	0.33	47.1	12
PPE60_A	68.8	0.51	52.9	20
PPE20 B	66.7	0.35	64.7	28
PE31_A	64.7	0.4	52.9	20
PE18_A	62.5	0.22	58.8	36
PPE69_B	62.5	0.30	36.4	4
PPE26_B	56.3	0.20	58.8	29
PPE69_D	53.3	0.27	70.6	48
PPE69_C	52.9	0.34	58.8	20
PPE65_B	50.0	0.14	ND	ND
PPE60_B	47.1	0.26	64.7	32
PPE69_E	47.1	0.26	58.8	32
PPE60_C	46.7	0.36	41.2	12
PPE47_A	43.8	0.20	ND	ND
PPE18_D	41.2	0.22	5.9	0
PE15 A	41.2	0.34	17.6	8
PPE26_C	41.2	0.13	35.3	8
PE20_A	41.2	0.14	5.9	0
PPE31_B	41.2	0.23	ND	ND
PPE46_B	40.0	0.37	ND	ND
PPE60_D	35.3	0.25	35.3	5
PPE18_E	29.4	0.14	17.6	4
PPE26_E	26.7	0.11	11.8	0
PPE60_E	23.5	0.25	47.1	16
PE36_A	13.3	0.04	52.9	20
PPE69_A	13.3	0.05	41.2	12
PPE26_D	5.9	0.01	0.0	0
ESX	100.0	2.21	70.6	56

**a.** Individual cattle samples were scored as positive if ΔOD_450_ values were ≥0.1, and human samples were scored as positive if ≥20 SFC were counted per 10^6^ PBMC.

Bioinformatic, phylogenetic and functional analyses have revealed a close association between PE/PPE proteins and the ESX regions [19]. Recent evidence suggests that several PE/PPE proteins are secreted by the ESX apparatus [23], [24], [25], which may render them more likely to be immunogenic. We compared the recognition of PE/PPEs originating from different ESX groupings (Figure S1, Table 1), to determine whether these were differentially immunogenic. Peptide pools representing the PE/PPE proteins encoded by partial *esx* clusters were significantly more immunogenic, with 23/27 (79.3%, p  = 0.0096, Fisher’s exact test) of these eliciting positive responses in 2 or more infected cattle compared to peptide pools representing isolated PE/PPE pairs and complete ESX clusters (56.7%, 42/74). These results indicate that genomic association with a complete *esx* cluster is not a prerequisite for immune recognition.

### *M. tuberculosis*-infected Humans Demonstrate a Similar PE/PPE Immune Recognition Hierarchy to *M. bovis-*infected Cattle

We performed a second cross-sectional study to assess PE/PPE immunogenicity in humans with active *M. tuberculosis* infection. For this, we selected 21 pools that were recognized in ≥40% cattle, plus an additional 7 pools corresponding to adjacent PE proteins or downstream pools from the same PPE protein (Table 2). Once again, a striking number of immunogenic pools were identified. A total of 14/28 pools were recognized in ≥50% individuals. Ten individuals (58.8%) recognized ≥10 pools each (mean number of pools recognized, 12.0; SD, 8.3; range, 22).

**Figure 2:**
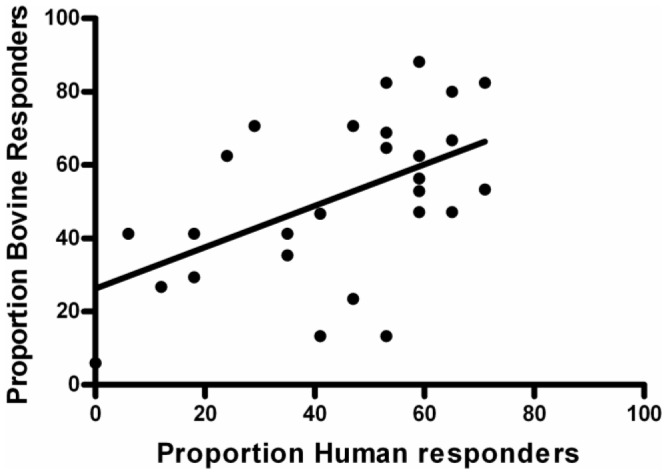
Comparison of proportion responders in human and bovine hosts with active TB. Responses to 28 PE/PPE peptide pools were assessed in both cattle and humans with active TB, by Bovigam or ELISPOT assays. Individual cattle samples were scored as positive if ΔOD_450_ values were ≥0.1, and human samples were scored as positive if ≥20 SFC were counted per 10^6^ PBMC. Linear regression analysis produced the line shown. A Pearson correlation test revealed a statistically significant correlation between the proportion human responders and the proportion bovine responders; r(26)  = 0.539, 2-tailed p value  = 0.0031.

We then compared immune recognition in the two species. This revealed a similar immune recognition hierarchy in cattle and humans exposed to *M. bovis* and *M. tuberculosis*, respectively (Figure 2, Table 2). Ten pools were recognized in ≥50% of individuals for both species. There was a statistically significant correlation between responses in humans and cattle, both as measured by proportion responders (Figure 2) and mean responses (data not shown).

### Disease Status does not Alter the PE/PPE Immune Recognition Hierarchy

**Figure 3:**
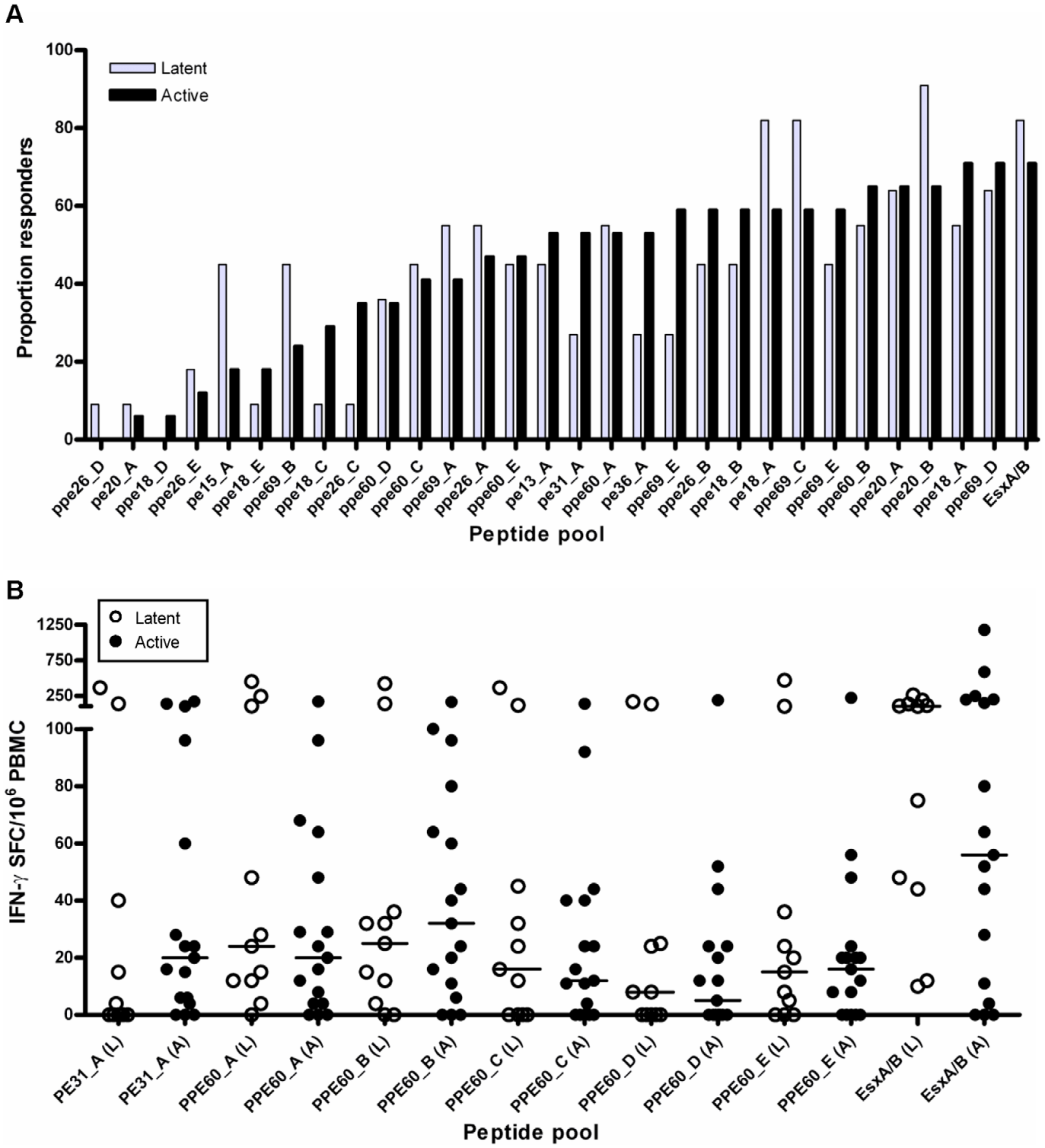
Immune responses to PE/PPE proteins in humans with active and latent infection. PBMC from *M. tuberculosis*-sensitized individuals were stimulated with PE/PPE peptide pools, and IFN-γ producing cells were enumerated by ELISPOT. (A) Proportion responders to all PE/PPE pools analyzed. Grey bars, individuals with latent infection; Black bars, individuals with active infection. (B) Number of IFN-γ producing cells measured after stimulation with PE/PPE peptide pools. White symbols, individuals with latent infection; Black symbols, individuals with active infection. No statistically significant differences were observed between responses in individuals with latent and active infection for any of the peptide pools analyzed (as determined by Mann Whitney U test). Results for a representative set of pools shown, horizontal lines indicate median responses.

The results described above revealed a very similar PE/PPE immune recognition hierarchy in humans and cattle with TB. However, in both species, samples were obtained from individuals with active disease, which could bias responses towards a particular immune profile. We therefore determined whether differences in disease status or stage impacted on the immune response to these proteins. We first compared immune responses to PE/PPE peptide pools in humans with active and latent infection. Overall, a similar PE/PPE immune hierarchy was observed for these 2 groups (Figure 3A). No statistically significant difference was observed between the magnitude of IFN-γ responses of active and latent cases for any of the PE/PPE pools tested (Figure 3B and data not shown).

**Figure 4:**
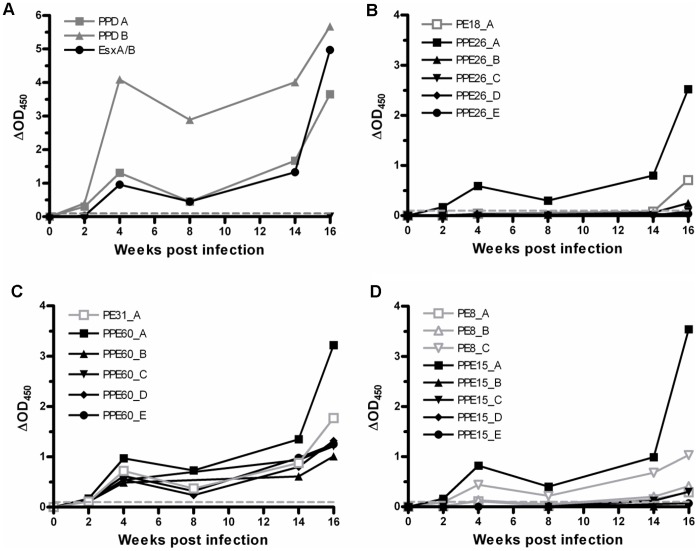
Temporal profile of bovine IFN-γ responses to PE/PPE peptide pools. IFN-γ responses were measured following stimulation of whole bovine blood with PE/PPE peptides and control antigens before, and at multiple time points following, experimental infection of cattle with *M. bovis* AF2122/97. (A) Responses to controls antigens; avian PPD (PPD-A), bovine PPD (PPD-B) and an EsxA/B peptide cocktail. (B) to (D) Representative responses to sets of PE/PPE peptide pools. Open grey symbols, PE pools; Solid black symbols, PPE pools; ▪, ▴, ▾, ♦, •, pools A-E, respectively (B) IFN-γ responses to PE18/PPE26 peptide pools. (C) IFN-γ responses to PE31/PPE60 peptide pools. (D) IFN-γ responses to PE8/PPE15 peptide pools. Mean responses for a single representative animal are shown.

To further assess the impact of disease stage on PE/PPE immune recognition, we performed a prospective study to examine the kinetics of immune responses to PE/PPE peptide pools over a 16 week course of infection in cattle. Representative data is shown for one animal in Figure 4. From 2–3 weeks post-infection onwards, positive responses to the control antigens PPD-B, PPD-A and the EsxA/B peptide cocktail were detected (Fig 4A). As we have previously observed [31], [32], the magnitude of IFN-γ responses fluctuated over time, likely reflective of the dynamic nature of the circulating peripheral immune cell population. Responses to PE/PPE peptide pools followed similar kinetics to responses to control antigens. Representative response profiles are shown for peptide pools representing 3 PE/PPE pairs in Figure 4B-D. In total, for the animal shown in Figure 4, 20/101 peptide pools were recognized as early as 2 weeks post-infection (Figure 4B-D and data not shown). An additional 15 peptide pools were recognized at 4 weeks post-infection. Responses to the 35 recognized peptide pools were then sustained at subsequent time points (14 and 16 weeks post-infection). It therefore appears that under the experimental conditions employed, the set of PE/PPE pools which is recognized does not substantially alter over the course of the infection. A similar trend was observed for an additional 4 animals tested (data not shown); once a PE/PPE peptide pool was recognized, the response towards that pool was observed at all later time points. In addition, a similar immune recognition profile was observed in naturally infected cattle (data not shown, Table S2). In summary, recognition of particular PE/PPE proteins occurred early and was sustained throughout the course of infection as well as in different disease statuses.

### Conserved N-terminal Domains are More Immunogenic

**Figure 5:**
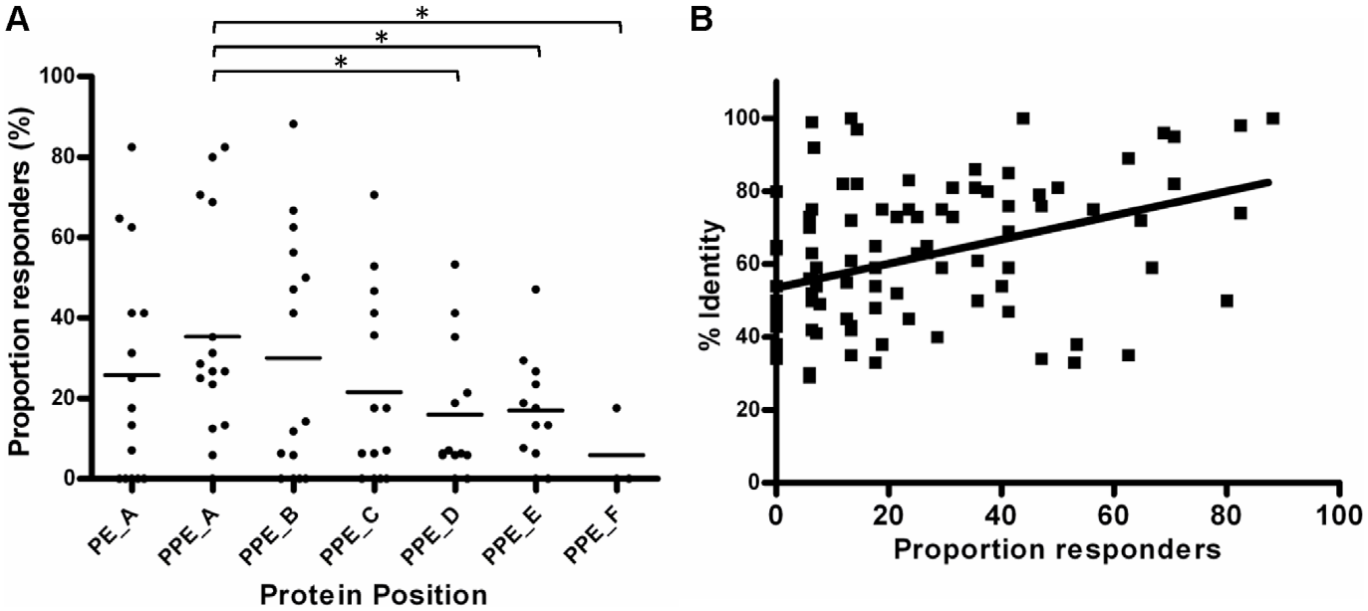
Immune recognition correlates with sequence conservation. (A) IFN-γ responses were measured following stimulation of whole bovine blood with PE/PPE peptide pools, and scored as positive if ΔOD_450_ values were ≥0.1. The proportion of responders was plotted along the length of the PE/PPE proteins, where A corresponds to the peptide pool closest to the N-terminal. *, 2-tailed p value <0.05 (unpaired t-test). The letters A to F denote the position of the peptide pool across the length of the protein, with A nearest the N-terminal. PE_A represents the conserved 110 aa region, while PPE_A and PPE_B encompasses the conserved 180 aa region of the PPE proteins. (B) The proportion responders to PE/PPE peptide pools was plotted against % identity, which corresponds to the % identity of the amino acid sequence corresponding to that pool to its closest match in the *M. tuberculosis* genome sequence. Linear regression analysis produced the line shown. A Spearman correlation test indicated revealed a statistically significant correlation between % identity and proportion bovine responders; rho(98)  = 0.401, 2-tailed p value <0.0001.

Analysis of bovine and human immune responses to PE/PPE peptide pools highlighted a trend towards better recognition of N-terminal peptide pools. For example, in the 3 representative examples shown in Figure 4 B–D, N-terminal peptide pools elicited larger IFN-γ responses, as measured by ΔOD_450_. We examined this further by plotting combined data for all PE and PPE pools across the length of the proteins. The shorter PE proteins were represented by a single pool, whereas the longer PPE proteins were represented by up to 6 pools. The immunogenicity of the peptide pools both as measured by the proportion responders (Figure 5A) and magnitude of responses (data not shown), decreased across the length of the protein. As previously noted, the N-terminal regions of the PE/PPE proteins are relatively conserved. We observed a statistically significant correlation between the degree of sequence conservation and immune recognition (Figure 5B). This data therefore indicates that the most conserved regions of these proteins are most likely to elicit an immune response.

### Antigenic Cross-reactivity is Associated with Immune Recognition

The results described above suggested that the immune responses observed were associated with immunological cross-reactivity towards the relatively conserved PE/PPE N-terminal domains. To examine in detail whether responses were directed towards epitopes situated in the most conserved regions, we characterized responses to individual peptides within selected pools. We first measured responses to 258 constituent peptides from 26 pools that had elicited positive responses in cattle. Responses to individual peptides were examined in up to 5 animals. For all pools, at least 1 peptide that elicited a positive response was identified. Up to 8 (median  = 2.5) peptides/pool were recognized. A total of 97 individual positive peptides were identified (Table S1).

**Figure 6:**
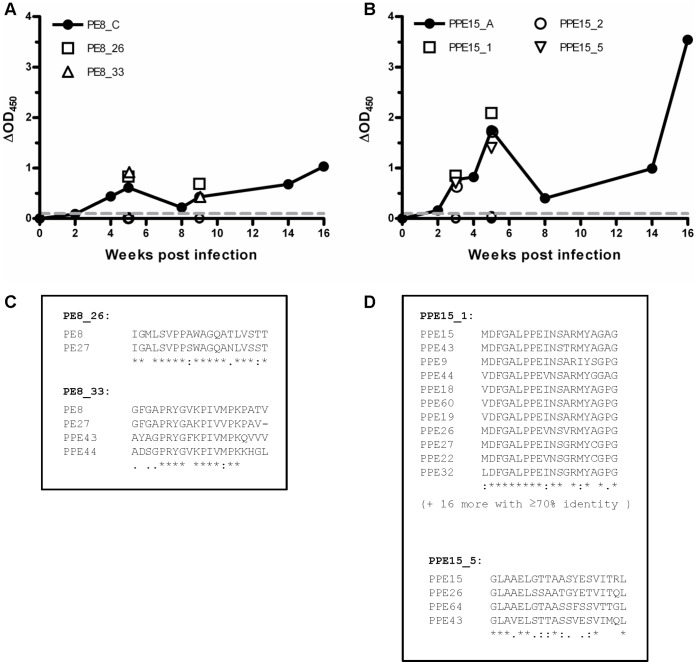
Whole blood IFN-Υ responses to individual and pooled PE/PPE peptides. Peptide pools eliciting positive responses were further analysed by assessing the contribution of each constituent peptide at 3, 5 or 9 weeks post-infection**.** Representative data are shown for peptides corresponding to (A) One pool derived from PE8, and (B) One pool derived from PPE15. The solid lines represent the temporal response to a peptide pool, while individual symbols correspond to individual peptides. (C) and (D) BLASTP homology searches were performed with peptides which elicited positive responses, and closest matches are shown in a CLUSTALW alignment.

To assess the extent of potential cross-reactivity, sequence comparisons between the peptides eliciting positive responses and other PE/PPE proteins were performed. This underscored the tendency for recognition of the most conserved peptides, which could potentially harbor cross-reactive epitopes. As illustrated in Figure 6, the peptides which elicited positive responses showed >70% amino acid identity to at least one, and frequently multiple, independent PE/PPE proteins. This trend was typical of other peptides examined, with 70 of the 97 positive peptides showing >70% amino acid sequence homology to 1 or more additional PE or PPE regions (Table S1). This suggests that the immune response to these peptides is partly attributable to recognition of cross-reactive epitopes.

**Figure 7:**
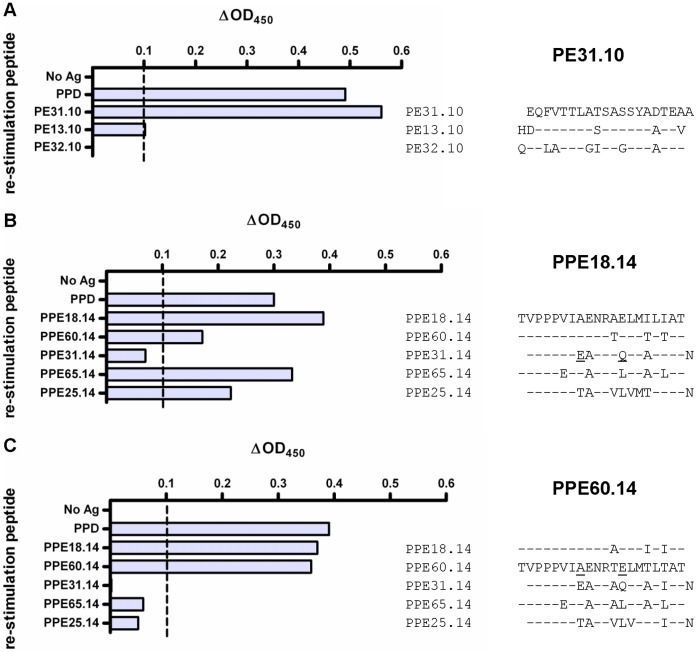
IFN-γ responses of short-term T cell lines to potentially cross-reactive PPE peptides. Short-term T cell lines were raised by primary stimulation of bovine PBMC with individual PE or PPE peptides in the presence of IL-2. Following initial expansion with (A) PE31.10, (B) PPE18.14 or (C) PPE60.14, cells were re-stimulated with selected peptides, predicted to be immunologically cross-reactive. IFN-γ production was then measured using the ELISA-based Bovigam assay. The amino acid sequence of the initial stimulation peptide is shown in alignment with restimulation peptides, with amino acid substitutions indicated. Responses are shown as ΔOD_450_ values, and the dashed line indicates the positivity cut-off value of 0.1.

A high degree of sequence identity alone does not necessarily guarantee T cell cross-reactivity, and even single amino acid substitutions can alter MHC binding or T cell recognition of the epitope. Therefore, we further examined the functional consequences of sequence variation in different PE/PPE proteins in short-term bovine T cell lines. In these experiments, we performed an initial expansion of antigen-specific T cell clones with a selected peptide in the presence of IL-2. The expanded clones were restimulated either with the original peptide, or with a second non-identical, but similar, peptide. Responses were then assessed by measuring IFN-γ production. In these experiments, if T cell cross-reactivity occurred, we would expect non-identical, but similar, peptides to elicit IFN-γ production upon secondary stimulation of the T cell lines. In the first example, T cells were expanded by primary stimulation with peptide PE31_10 (Figure 7A). As expected, IFN-γ was produced upon restimulation with this peptide. In contrast, no detectable IFN-γ was produced upon restimulation with PE32_10, which shares 13 residues with PE31_10. However, restimulation with PE13_10 (which has 4 amino acid differences when compared to PE31_10) elicited a detectable response. This indicates that there is cross-recognition of PE13_10 by the PE31_10-expanded T cell population.

Cross-reactivity was further assessed for PPE-expanded T cell lines, and we observed similar results for T cell lines established by primary stimulation with either PPE18_14 or PPE60_14 (Figure 7B and 7C, respectively). These peptides are themselves 85% identical, and PPE18_14-expanded T cell lines responded to PPE60_14 and vice versa. PPE18_14-expanded lines also responded to the corresponding peptides from PPE65 (PPE65_14, 15/20 residues identical) and PPE25 (PPE25_14, 12/20 residues identical). Stimulation with PPE31_14 elicited low IFN-γ responses in PPE18_14-established cell lines (Figure 7B), and no detectable response in PPE60_14-expanded lines (Figure 7C), suggesting that critical MHC-binding or T cell recognition residues were altered. Importantly, despite multiple amino acid changes, 3 out of 4 peptides derived from PPE proteins other than PPE18 elicited IFN-γ responses above the cut-off of 0.1 for PPE18_14-derived lines. This supports the interpretation that the recall immune responses observed in this study are partially driven by cross-reactive T cells.

## Discussion

To address gaps in our understanding of the PE/PPE proteins and their role in infection, we examined immune responses to a large subset of PE/PPE proteins. The 36 proteins selected for this study represented a diverse cross-section of the PE/PPE protein families. We assessed PE/PPE immunogenicity in two host species, namely cattle infected with *M. bovis* and humans infected with *M. tuberculosis*. These both represent mycobacterial infections in natural hosts, and offer the opportunity to investigate immune responses to mycobacterial antigens. We first performed high-throughput screening of infected cattle for immunological responsiveness to 1043 peptides representing 36 PE/PPE proteins. A striking number of peptide pools elicited IFN-γ production, indicating sensitization of the cattle to PE/PPE proteins. In total, peptides representing 9 PE/PPE proteins elicited positive responses in ≥50% of infected cattle. Seven of these had not been previously described in the literature. This work therefore substantially advances our knowledge of the high immunogenic potential of the PE/PPE proteins.

A subset of peptide pools was assessed in human TB patients, and confirmed the highly immunogenic nature of these proteins. Furthermore, this revealed a very similar hierarchy of immunodominance (where this refers to peptide pools that are most frequently recognized) in bovine and human hosts. Although PE/PPE proteins are considered to be among the most variable members of the mycobacterial proteome, it should be noted that it is predominantly the PE-PGRS and PPE-MPTR family members that this applies to, and the subset of PE/PPE proteins selected for this study were in fact relatively conserved. Twenty-three of the 36 proteins examined here were identical in *M. bovis* AF2122/97 and *M. tuberculosis* H37Rv. For the remainder, the differences observed were predominantly single point mutations. Similar results were observed for 27 clinical isolates of *M. tuberculosis* for which complete genome sequences were available (data not shown). It could therefore be argued that a similar immune recognition hierarchy is unsurprising. Nonetheless, 2 recent publications reported that several *pe/ppe* genes are differentially transcribed in *M. bovis* and *M. tuberculosis* [33], [34], 6 of which were included in this study. This, together with the differing host tropisms of the 2 species could suggest that differential expression and/or recognition of PE/PPE proteins might occur. Despite this, we still observed a similar immune recognition hierarchy.

The initial part of this study was performed in cattle and human hosts with active TB, which could imply that the observed hierarchy of immune recognition is only reflective of uncontrolled infection. However, similar results were obtained when we compared immune responses in humans with active or latent infections, in naturally infected cattle (Table S2) and at multiple time points over a 4-month course of disease in experimentally infected cattle. This indicates that numerous PE/PPE family members are expressed and recognized throughout infection, potentially playing an important functional role.

The immune recognition data presented here raises questions regarding the mechanisms underlying differential PE/PPE immunogenicity. One simple explanation could be that there is differential expression of PE/PPE family members during infection. At the mRNA level, there are indications of differential *pe/ppe* gene expression *in vitro* [35]. However, *in vivo* analysis is more challenging. Analysis of transcriptional profiles of *M. tuberculosis* isolated from human pulmonary tissue demonstrated that several PE/PPE proteins are indeed upregulated upon infection [36], [37], [38], but there is no clear correlation between these and the immunogenic rank of antigens identified in this study. mRNA levels do not necessarily translate to antigen abundance, and methods for measuring *in vivo* protein levels are technically limited. One recent study attempted to characterize the *in vivo M. tuberculosis* proteome by large-scale LC-MS [39]. Interestingly, this approach identified several of our top-ranking PE/PPE proteins in guinea pig lungs at 90 days post-infection, suggesting that immunogenicity could in part be enhanced by higher protein expression levels. However, this proteomic approach is not comprehensive and further investigation will be required to determine whether there is a direct correlation between protein abundance and immunogenicity.

Previous studies have shown that secreted proteins tend to be immunogenic [40]. Several PE/PPE proteins are known to be secreted [10], and there are indications that this could be ESX-mediated [23], [24], [25], [26], [41]. We recently assessed the immunogenicity of a panel of predicted secreted antigens, including EsxB/EsxA orthologues [41]. The top-ranking antigens identified in that study included EsxA and/or EsxB homologues from 2 complete and 4 partial *esx* clusters. In the present study, pools representing the PE/PPE proteins encoded upstream of these *esxA/esxB* orthologues were recognized in ≥30% of infected cattle. Furthermore, we found that PE/PPE proteins encoded by partial ESX clusters were significantly more immunogenic than those from complete ESX clusters. As previously noted, this indicates that association with a complete ESX cluster is not required for immune recognition. As the complete ESX cluster is expected to be necessary for secretion [42], this raises the interesting possibility that there could be cross-talk between ESX clusters, with the secretion of PE/PPE proteins mediated by ESX clusters encoded at genomically distal locations.

Further factors which could influence PE/PPE immunogenicity are MHC binding and T cell recognition of the MHC II-peptide complex. Bioinformatic analysis revealed no particular bias toward higher epitope density in the more immunogenic peptide pools (data not shown). However, the accuracy of current MHC II-binding prediction methods is limited, and does not take into account T cell recognition. Although experimental assessment of MHC II binding was beyond the scope of this study, we note that a large proportion of peptide pools were recognized in hosts from 2 different species and with diverse MHC repertoires. This indicates that the PE/PPE peptide library examined could include several promiscuous epitopes, and suggests that these may be promising vaccine candidates.

This study suggests that similar PE/PPE family members are expressed and recognized at different stages of infection. Even if different members of this subset were expressed at different time points, our results indicate that cross-reactive, PE/PPE-directed T cell populations are present throughout infection. As T cell responses appear to be primarily targeted towards the most conserved regions of the PE/PPE proteins, these would be expected to recognize multiple PE/PPE family members. The circulating T cell population might be slower to respond (i.e. antigen-specific effector cells could still be present even if the particular antigen is no longer expressed), and subtle changes may not be detected with the approaches used in this study. However, previous work suggests that T cell responses are reflective of antigen load [43], and would be expected to wane in the absence of a particular antigen. In contrast, we observed increasing magnitudes of T cell responses at later time points, providing an indirect indication that the antigens are still being expressed.

This work suggests that immunological cross-reactivity occurs in a significant proportion of the observed PE/PPE immune responses. We showed that peptide pools corresponding to the most conserved regions of the proteins are most likely to elicit IFN-γ production. This is best illustrated by the example of PE32/PPE65, for which the genes are deleted in *M. bovis.* However, PPE65 shows a high degree of similarity to several other PPE proteins, and one peptide pool (PPE65_B) derived from this protein is recognized by 50% of bovine hosts (Table 2, Table S1). Furthermore, at the individual peptide level, peptides that elicited positive responses also demonstrated high degrees of sequence conservation. The idea that immune recognition is reflective of cross-reactivity was further corroborated with short-term bovine T cell lines raised by primary stimulation with a particular PE or PPE peptide. These produced IFN-γ in response to stimulation with peptides corresponding to other PE/PPE proteins, highlighting that even altered amino acid sequences were able to elicit recall responses. Together, these results suggest that even if different PE/PPE variants were expressed, this would likely be an ineffective immune escape mechanism, due to extensive T cell cross-reactivity.

A recent report which combined analysis of multiple genome sequences with immunogenicity data demonstrated an unexpectedly high level of conservation of human T cell epitopes [44]. Unfortunately, due to inherent technical limitations of the sequencing methodology, PE/PPE proteins were excluded from that analysis. While it is possible that some members of these protein families may prove an exception to that finding, the subset studied here appears to be relatively conserved both within and between species. The work described here adds another dimension and shows that even when PE/PPE amino acid sequences do vary, this does not necessarily impair epitope recognition. This suggests that even the unexpectedly high degree of epitope conservation calculated by Comas *et al* [44] may underestimate conservation at the level of the immune response.

A limitation of this study is that we have not assessed immune responses to any representatives of the PE_PGRS or PPE_MPTR subgroups. These family members possess highly repetitive domains that exhibit substantial genetic variation [3], [12], [45], [46], [47], and may be differentially recognized. It has previously been demonstrated that the conserved PE domain of PE_PGRS33 elicited primarily Th1-type responses [48], and we recently showed that T cell responses were predominantly directed towards the non-conserved region of PE_PGRS62 (Rv3812) [49]. This underscores the complexity of the immune response to these antigens and suggests that further systematic analysis of immune responses to these PE/PPE subgroups will be required to establish the functional and immunological consequences of the observed variation within their C-terminal domains. In future work, it would be of interest to perform a comprehensive analysis of these subgroups.

Measuring IFN-γ production alone provides a relatively isolated assessment of the immune response. In future work, a more detailed phenotypic characterization of responding cell populations and measurement of cytokines other than IFN-γ should be undertaken. Together with systematic, high-throughput analysis of responses to other PE/PPE family members, such as those from the PE_PGRS and PPE_MPTR subsets, this could help to unravel the cumulative impact of PE/PPEs on host immune responses.

As discussed by others [44], [50], immune recognition and subsequent host responses are not necessarily detrimental, and may indeed be beneficial, for pathogenic mycobacteria. In this study, PE/PPE proteins were recognized at all stages of disease, including in individuals with asymptomatic infections. This suggests that they provide some benefit for the bacterium to balance the cost of maintaining these large gene families. One possibility is that the robust T cell responses they elicit could promote tissue damage and subsequent transmission. An alternative hypothesis is that PE/PPE proteins could act as decoy antigens, overwhelming and mis-directing immune responses, thereby preventing the priming of potentially protective T cell populations. A further possibility is that these proteins play some other, yet-to-be elucidated, role in mycobacterial pathogenesis.

In summary, we report here on the most comprehensive analysis of PE/PPE immunogenicity to date, in two different host species. The 36 PE/PPE proteins examined here were selected from 4 different phylogenetic sub-groups, and therefore represented a large cross-section of the two protein families. We have shown that the PE/PPE proteins are highly immunogenic, and demonstrate a similar immune recognition hierarchy in different species and at different stages of infection. This work shows that PE/PPE-specific immune responses are largely driven by cross-reactivity. Some of the antigens that have been newly identified here could be useful as future vaccine candidates. However, any such initiatives should be accompanied by work to improve our understanding of which epitopes elicit protective immune responses, rather than inadvertently providing further benefit to the bacterium.

## Methods

### Ethics Statement

The study was approved by the University of Cape Town Research Ethics Committee (REC249/2009) and written informed consent was obtained from all human study participants. All bovine work was carried out in accordance with the UK Home Office Animal (Scientific Procedures) Act 1986, following approval by the AHVLA Ethical Review Board and the UK Home Office.

### Peptide Design, Synthesis and Preparation

36 PE/PPE proteins associated with, or duplicated from ESX clusters (Figure S1, [19]) were selected for study. *Mycobacterium tuberculosis* H37Rv amino acid (aa) sequences were used to design a library of 20 aa long peptides with a 12 residue overlap. *In silico* analysis was performed to identify duplicate peptides; exact duplicates were eliminated (Table S1), resulting in a final set of 1043 peptides. Peptides were prepared using multirod peptide synthesis (Pepscan systems, The Netherlands), and subjected to HPLC/MS to confirm purity. Lyophilized peptides were resuspended in Hanks balanced salt saline solution (HBSS) with 10% DMSO at 4 mg/ml. Peptides were used in assays at 5 µg/ml, either as individual peptides or in pools of 8–12 peptides per pool. Peptides from ESAT-6 (EsxA) and CFP-10 (EsxB) were synthesized by conventional solid-phase synthesis technology, and the EsxA/B peptide cocktail was prepared as previously described [51].

### Experimental Animals

Approximately six month-old male Holstein or Holstein cross calves were obtained from herds free of bovine tuberculosis (BTB), and were housed in appropriate biological containment facilities at AHVLA. Calves (n  = 17) were infected via the intratracheal route with 3×10^3^ CFU *Mycobacterium bovis* field isolate AF2122/97. Infection status was confirmed by single intradermal comparative cervical test (SICCT, [52]) and *in vitro* IFN-γ release assays. In addition, all animals presented with visible lesions and were culture positive when post-mortem examinations were carried out at approximately 4 months post-infection. Heparinized blood samples were collected before and at multiple time points following infection.

### Bovigam IFN-γ Release Assay

Bovigam (Prionics AG, Switzerland) assays were performed according to the manufacturer’s instructions. Briefly, heparinized bovine blood was collected, and whole blood cultures were initiated on the day of sampling. Blood cells were stimulated with control antigens or peptides for 48 h. Controls included: no antigen, 10 µg/ml of avian or bovine purified protein derivative (PPD) (Veterinary Laboratories Agency (VLA), United Kingdom) and 1 µg/ml pokeweed mitogen (SIGMA). Peptides were used at a final concentration of 5 µg/ml for each peptide. Following stimulation, plasma supernatants were harvested and stored at −80°C until further processing. IFN-γ levels were quantified using an ELISA-based assay performed with reagents supplied with the kit. As for previous studies [40], [41], [51], results were scored as positive if the optical density at 450 nm (OD_450_) minus the OD_450_ without antigen was ≥0.1 in both of the duplicate wells.

### Short-term Bovine T cell Lines

Peripheral blood mononuclear cells (PBMC) were isolated from heparinized bovine blood by Histopaque-1077 (SIGMA) density gradient centrifugation. PBMC were washed in HBSS containing heparin, then in RPMI 1640 tissue culture medium (Life Technologies, United Kingdom) supplemented with 10% fetal calf serum (SIGMA), nonessential amino acids (SIGMA), 5×10^−5^ M β-mercaptoethanol, 100 U/ml penicillin, and 100 µg/ml streptomycin sulphate (subsequently referred to as RPMI complete). Isolated PBMC were then used to set up short-term T cell lines or cultured antigen presenting cells (APC). APC were prepared by setting up 96 well plates with 1×10^6^ cells/well, then incubating at 37°C, 5% CO_2_ for 2 hours. Plates were washed 3 times with HBSS to remove non-adherent cells. The remaining adherent cells were incubated with RPMI complete at 37°C, 5% CO_2_ for 15 days, and then used for secondary antigen stimulation with cultured T cell lines. To generate T cell lines, PBMC were added to 24 well plates at 1×10^6^ cells/well. PBMC were incubated with 5 µg/ml primary peptide for 4 days, then with 10 U/ml Human IL-2 (Sigma) for a further 7 days to expand responsive clones. Cultures were weaned of IL-2 over a period of 4 days, then cells were harvested, washed and added to APC plates at 2×10^4^ cells/well. The secondary peptides were added at 5 ug/ml, and cultures were incubated at 37°C, 5% CO_2_ for 2 days. Cell-free supernatants were harvested and IFN-γ levels were quantified using the Bovigam kit as described above.

### Human Subjects

Samples were obtained from persons older than 21 years attending the Voluntary Counselling and Testing (VCT) services run by the Ubuntu clinic in Khayelitsha site B. Khayelitsha is a peri-urban township near Cape Town with a population of over 400,000 and an exceptionally high burden of TB (>1500 per 100,000 in 2005). Study groups were either (i) HIV seronegative persons with culture confirmed active TB (n  = 17) or (ii) HIV seronegative asymptomatic people (n  = 11) with skin test or *in vitro* evidence of latent TB infection (LTBI), where LTBI is defined as a Mantoux test of >10mm as per national guidelines PLUS a positive reaction to either CFP-10 or ESAT-6 in overnight ELISpot analysis. Peripheral blood mononuclear cells (PBMC) were extracted from heparinized whole blood within four hours of collection. PBMC were separated from the whole blood by Ficoll-Paque™ gradient technique and stored in liquid nitrogen until further analysis.

### ELISpot

The ELISpot assay was performed using the Mabtech ELISpot^PRO^ kit for human IFN-γ kit according to manufacturer’s instructions. Viable PBMC (2.5×10^5^ cells/well) were resuspended in RPMI with 10% Fetal calf serum and added to the pre-coated and blocked plates and stimulated with antigens and controls for 16–20 hours at 37°C with 5% CO2. After incubation, the plates were developed and the number of spots were counted using an ImmunoSpot 3.2 reader and verified manually. Controls included monoclonal anti-CD3 provided with the kit, and the EsxAB peptide cocktail as previously described [51]. A positive response was defined as ≥20 SFC/10^6^ PBMC above background.

### Statistical Analysis

Statistical analyses were performed using GraphPad Prism V4.0 software. The normality of the data was assessed by the D’Agostino and Pearson omnibus test. Non-parametric data was compared using the Mann-Whitney U test. Correlation between normally distributed data sets was analysed using the Pearson correlation test, while correlation between non-normally distributed data was analysed using the Spearman correlation test. P values <0.05 were considered to be significant.

## Supporting Information

Figure S1 Genomic arrangement of *pe/ppe* gene pairs associated with ESX gene clusters. *pe/ppe* gene pairs occur within (A) extended ESX regions, (B) upstream of *esat-6/cfp-10* homologues and (C) as isolated gene pairs. *pe* and *ppe* genes are indicated by light and dark blue arrows respectively. *cfp-10* and *esat-6* homologues are indicated by light and dark grey arrows, respectively. All other genes are indicated by white arrows. *pe/ppe* numbers and *esx* gene names are indicated to the left of each region, in the order in which they occur. Strikethrough  =  *pe/ppe* proteins not included in this study. Figure adapted from Gey van Pittius *et al.* (2006) *BMC Evol. Biol*.
(TIF)

Figure S2 Evolutionary relationships between the members of the PPE protein family. The phylogenetic tree was constructed from the phylogenetic analyses done on the 180 aa N-terminal domains of the PPE proteins. The tree was rooted to the outgroup, Rv3873 (PPE68), shown to be the first PPE insertion into ESX-1. Arrows indicate orthologues of genes present within the *M. smegmatis* genome sequence. Five sublineages (including the PPE-PPW, PPE-SVP and PPE-MPTR subfamilies) are indicated by Roman numerals. Grey highlights indicate the proteins selected for this study. Figure adapted from Gey van Pittius *et al.* (2006) *BMC Evol. Biol*.
(TIF)

Table S1 PE/PPE peptides used for stimulation. Provides peptide sequence, *M. bovis* sequence comparison and recognition data for individual peptides and pools.
(TIF)

Table S2 IFN-γ responses to PE/PPE peptide pools in naturally infected cattle. Whole blood from naturally infected cattle (single intradermal comparative tuberculin test (SICTT)-positive reactors from herds known to have bovine tuberculosis as determined by government veterinarians of the Animal Health Agency) was stimulated with pools of peptides representing 36 PE/PPE proteins. IFN-γ production was assessed by the ELISA-based Bovigam assay. IFN-Y responses are expressed as OD_450_– Nil antigen well reading (ΔOD_450_). Pink highlights indicate positive responses (≥0.1). Green highlights indicate pools eliciting positive responses in ≥60% experimentally infected cattle (for comparison). ND  =  not determined (as replicates deviated by >30%). Briefly, 5 out of the 8 field reactors demonstrated responses to multiple peptide pools. In those responding, between 8 and 40 pools were recognised per animal, with a total of 37 pools recognised in 2 or more animals. The small number of animals demonstrating positive responses precludes robust statistical analysis. However, there appears to be a similar trend in the set of pools that are recognised, and we note that of the 11 top-ranking pools (recognised in >60% of experimentally infected cattle), all but one were recognised in 2 or more of the naturally infected animals.
(TIF)
