# Supplementary figures and images for: Conserved Immune Recognition Hierarchy of Mycobacterial PE/PPE Proteins during Infection in Natural Hosts
Source: PLoS One. 2012 Aug 1;7(8):e40890. doi: 10.1371/journal.pone.0040890 (PMC3411574; doi:10.1371/journal.pone.0040890)

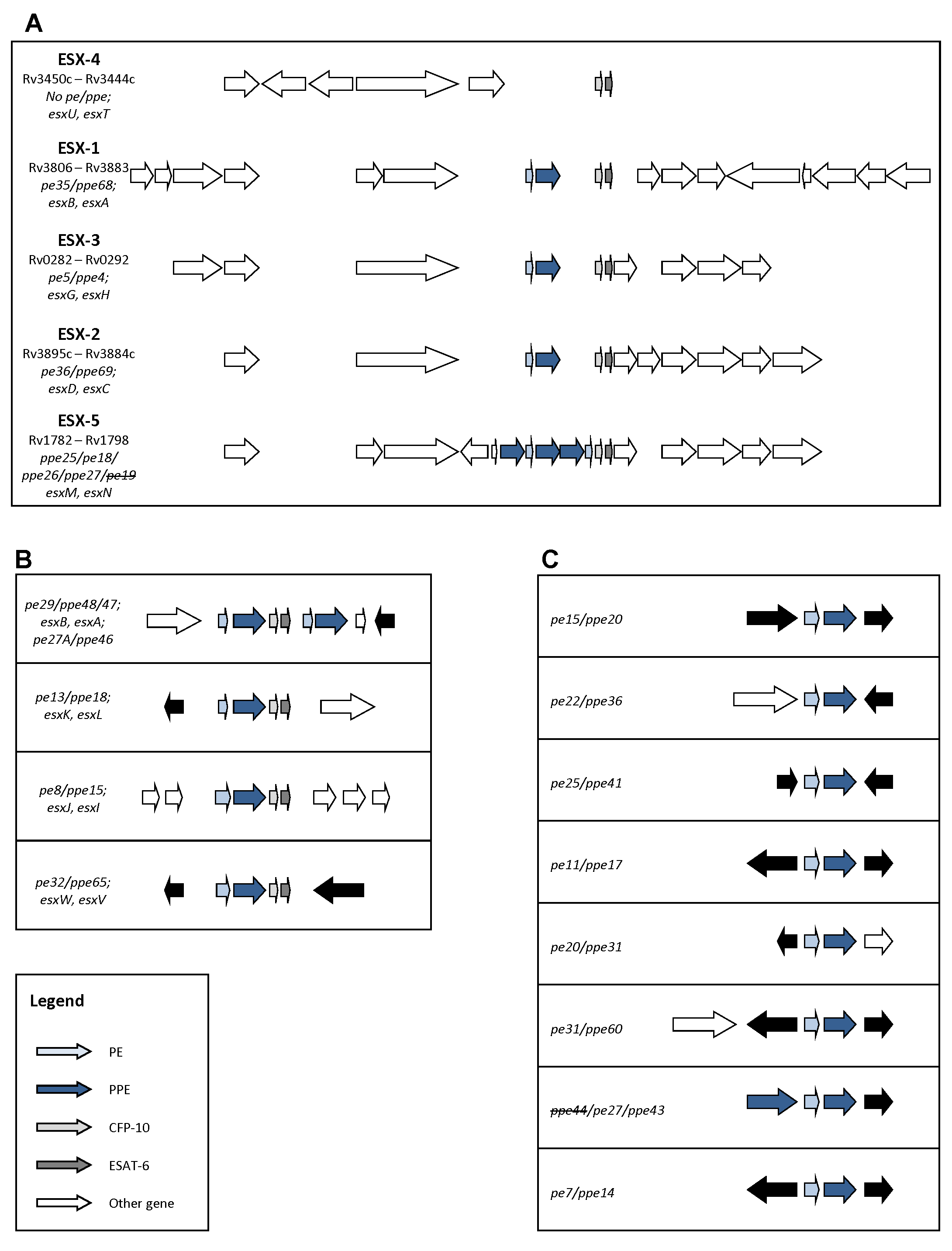

Supplement: Figure S1 — Genomic arrangement of pe/ppe gene pairs associated with ESX gene clusters. pe/ppe gene pairs occur within (A) extended ESX regions, (B) upstream of esat-6/cfp-10 homologues and (C) as isolated gene pairs. pe and ppe genes are indicated by light and dark blue arrows respectively. cfp-10 and esat-6 homologues are indicated by light and dark grey arrows, respectively. All other genes are indicated by white arrows. pe/ppe numbers and esx gene names are indicated to the left of each region, in the order in which they occur. Strikethrough = pe/ppe proteins not included in this study. Figure adapted from Gey van Pittius et al. (2006) BMC Evol. Biol. (TIF) [file pone.0040890.s001.tif]

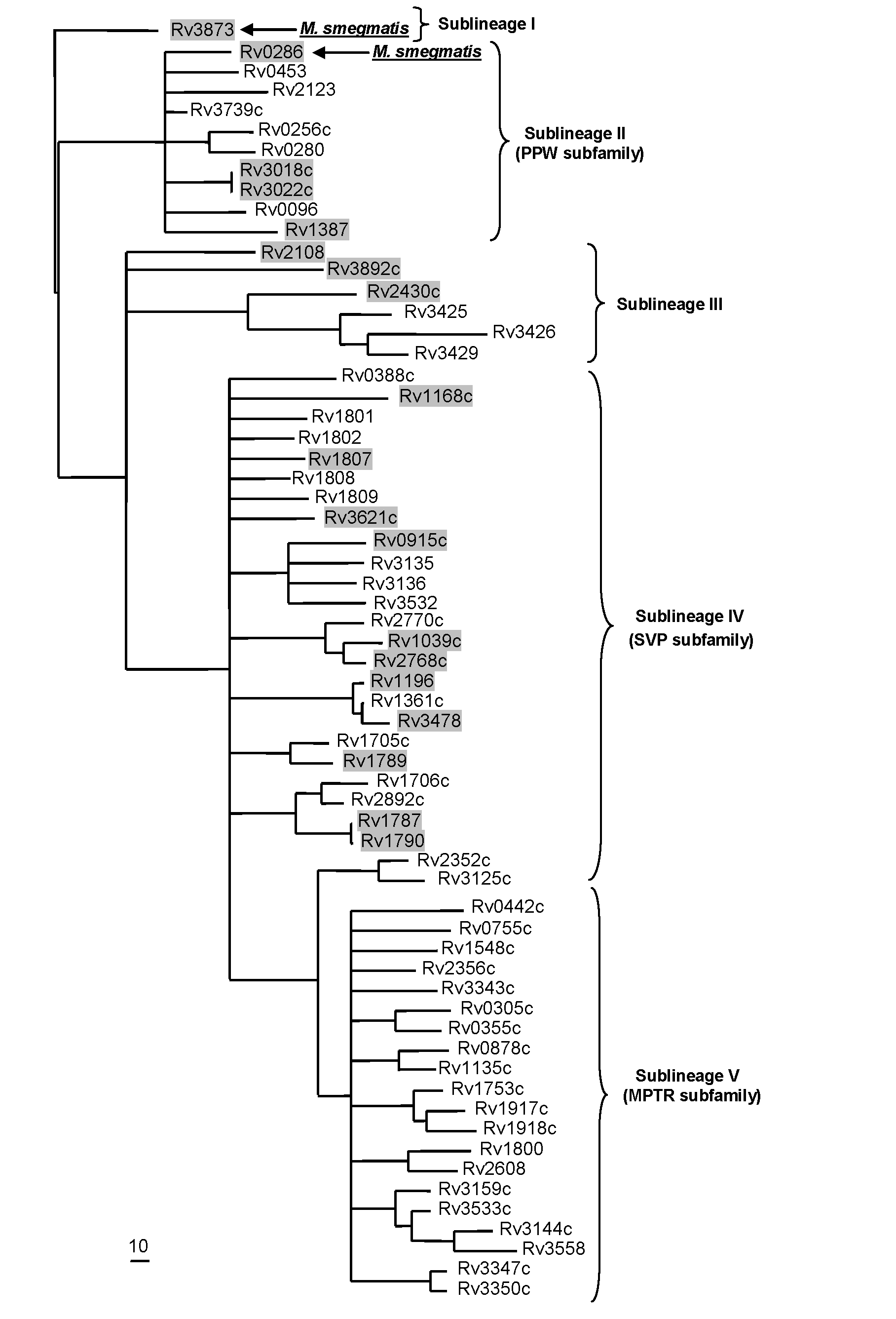

Supplement: Figure S2 — Evolutionary relationships between the members of the PPE protein family. The phylogenetic tree was constructed from the phylogenetic analyses done on the 180 aa N-terminal domains of the PPE proteins. The tree was rooted to the outgroup, Rv3873 (PPE68), shown to be the first PPE insertion into ESX-1. Arrows indicate orthologues of genes present within the M. smegmatis genome sequence. Five sublineages (including the PPE-PPW, PPE-SVP and PPE-MPTR subfamilies) are indicated by Roman numerals. Grey highlights indicate the proteins selected for this study. Figure adapted from Gey van Pittius et al. (2006) BMC Evol. Biol. (TIF) [file pone.0040890.s002.tif]
