# Supplementary material for: Conserved Immune Recognition Hierarchy of Mycobacterial PE/PPE Proteins during Infection in Natural Hosts
Source: PLoS One. 2012 Aug 1;7(8):e40890. doi: 10.1371/journal.pone.0040890 (PMC3411574; doi:10.1371/journal.pone.0040890)
Supplement: Table S2 — IFN-γ responses to PE/PPE peptide pools in naturally infected cattle. Whole blood from naturally infected cattle (single intradermal comparative tuberculin test (SICTT)-positive reactors from herds known to have bovine tuberculosis as determined by government veterinarians of the Animal Health Agency) was stimulated with pools of peptides representing 36 PE/PPE proteins. IFN-γ production was assessed by the ELISA-based Bovigam assay. IFN-Y responses are expressed as OD450– Nil antigen well reading (ΔOD450). Pink highlights indicate positive responses (≥0.1). Green highlights indicate pools eliciting positive responses in ≥60% experimentally infected cattle (for comparison). ND = not determined (as replicates deviated by >30%). Briefly, 5 out of the 8 field reactors demonstrated responses to multiple peptide pools. In those responding, between 8 and 40 pools were recognised per animal, with a total of 37 pools recognised in 2 or more animals. The small number of animals demonstrating positive responses precludes robust statistical analysis. However, there appears to be a similar trend in the set of pools that are recognised, and we note that of the 11 top-ranking pools (recognised in >60% of experimentally infected cattle), all but one were recognised in 2 or more of the naturally infected animals. (TIF) [file pone.0040890.s004.tif]

**Table S2. IFN-γ responses to PE/PPE peptide pools in naturally infected cattle.** Whole blood from naturally infected cattle (single intradermal comparative tuberculin test (SICTT)-positive reactors from herds known to have bovine tuberculosis as determined by government veterinarians of the Animal Health Agency) was stimulated with pools of peptides representing 36 PE/PPE proteins. IFN-γ production was assessed by the ELISA-based Bovigam assay. IFN-Y responses are expressed as OD_450_ – Nil antigen well reading (ΔOD_450_). Pink highlights indicate positive responses (≥0.1). Green highlights indicate pools eliciting positive responses in ≥60% experimentally infected cattle (for comparison). ND = not determined (as replicates deviated by >30%).

Briefly, 5 out of the 8 field reactors demonstrated responses to multiple peptide pools. In those responding, between 8 and 40 pools were recognised per animal, with a total of 37 pools recognised in 2 or more animals. The small number of animals demonstrating positive responses precludes robust statistical analysis. However, there appears to be a similar trend in the set of pools that are recognised, and we note that of the 11 top-ranking pools (recognised in >60% of experimentally infected cattle), all but one were recognised in 2 or more of the naturally infected animals.

| **Ag /**  **Animal** | **1** | **2** | **3** | **4** | **5** | **6** | **7** | **8** | **Total # positive** | **% responders amongst experimentally infected animals** |
| --- | --- | --- | --- | --- | --- | --- | --- | --- | --- | --- |
| PPD A | 0.94 | 0.30 | 0.20 | 0.13 | 0.19 | 1.65 | 2.01 | 0.27 |  |  |
| PPD B | 3.59 | 0.50 | 2.11 | 0.90 | 1.06 | 4.05 | 3.97 | 1.44 |  |  |
| SEB | 2.64 | 0.59 | 1.41 | 1.08 | 1.08 | 2.37 | 2.61 | 1.10 |  |  |
| ESX | 2.77 | 0.46 | 0.73 | 0.25 | 0.61 | 1.54 | 2.07 | 0.23 |  |  |
| PE35 A | 0.00 | 0.08 | 0.00 | 0.00 | 0.00 | 0.08 | 0.00 | ND | 0 | 0.0 |
| PPE68 A | 0.00 | 0.09 | 0.00 | ND | 0.01 | 0.01 | 0.00 | 0.00 | 0 | 0.0 |
| PPE68 B | 0.00 | 0.12 | 0.00 | 0.00 | 0.02 | 0.00 | 0.00 | ND | 1 | 0.0 |
| PPE68 C | 0.00 | 0.06 | 0.00 | ND | 0.00 | 0.00 | 0.01 | 0.01 | 0 | 0.0 |
| PPE68 D | 0.04 | 0.06 | 0.00 | 0.00 | 0.00 | 0.00 | 0.01 | ND | 0 | 0.0 |
| PE5 A | 0.03 | 0.07 | 0.00 | 0.00 | 0.00 | 0.00 | ND | ND | 0 | 0.0 |
| PPE4 A | 0.00 | 0.01 | 0.00 | 0.00 | 0.00 | 0.00 | 0.00 | 0.00 | 0 | 5.9 |
| PPE4 B | 0.02 | 0.07 | 0.00 | ND | 0.00 | 0.00 | 0.00 | ND | 0 | 5.9 |
| PPE4 C | 0.13 | 0.08 | 0.00 | 0.17 | 0.00 | 1.19 | 0.00 | 0.02 | 3 | 17.6 |
| PPE4 D | 0.00 | 0.06 | 0.00 | 0.00 | ND | 0.00 | 0.00 | ND | 0 | 5.9 |
| PPE4 E | 0.00 | 0.11 | 0.00 | 0.00 | 0.00 | 0.00 | 0.01 | ND | 1 | 17.6 |
| PPE4 F | 0.03 | 0.22 | 0.00 | ND | 0.00 | 0.00 | 0.01 | 0.02 | 1 | 17.6 |
| PE36 A | 0.07 | 0.03 | 0.00 | 0.04 | 0.00 | 0.00 | 0.04 | ND | 0 | 13.3 |
| PPE69 A | 0.09 | 0.04 | 0.00 | 0.03 | ND | 0.00 | 0.01 | ND | 0 | 13.3 |
| PPE69 B | 0.14 | ND | 0.00 | -0.01 | 0.00 | 0.00 | 0.12 | ND | 2 | 62.5 |
| PPE69 C | 0.10 | 0.08 | 0.00 | 0.04 | 0.00 | 0.11 | 0.10 | ND | 2 | 52.9 |
| PPE69 D | 0.10 | 0.13 | 0.00 | 0.06 | 0.00 | 0.07 | 0.00 | ND | 1 | 53.3 |
| PPE69 E | 0.07 | 0.15 | 0.01 | 0.09 | 0.00 | 0.08 | 0.00 | ND | 1 | 47.1 |
| PPE25 A | 0.00 | 0.12 | 0.00 | ND | 0.00 | 0.00 | 0.01 | 0.03 | 1 | 6.3 |
| PPE25 B | 0.05 | 0.14 | 0.00 | ND | 0.00 | 0.00 | 0.01 | 0.00 | 1 | 14.3 |
| PPE25 C | 0.11 | 0.07 | 0.00 | ND | ND | 0.00 | 0.00 | 0.01 | 1 | 6.7 |
| PPE25 D | 0.03 | ND | 0.00 | ND | 0.01 | 0.00 | 0.00 | 0.00 | 0 | 0.0 |
| PE18 A | 0.11 | 0.11 | 0.00 | 0.00 | 0.02 | 0.11 | 0.00 | ND | 3 | 62.5 |
| PPE26 A | 0.18 | 0.07 | 0.02 | 0.25 | ND | 1.09 | 0.01 | 0.06 | 3 | 70.6 |
| PPE26 B | 0.03 | 0.13 | 0.05 | ND | 0.04 | ND | 0.02 | 0.01 | 1 | 56.3 |
| PPE26 C | 0.00 | 0.09 | 0.01 | 0.00 | 0.04 | 0.00 | 0.00 | 0.01 | 0 | 41.2 |
| PPE26 D | 0.00 | 0.12 | 0.00 | 0.00 | 0.00 | 0.01 | 0.00 | ND | 1 | 5.9 |
| PPE26 E | 0.00 | ND | 0.00 | 0.01 | 0.05 | 0.02 | 0.13 | 0.00 | 1 | 26.7 |
| PPE27 A | 0.18 | 0.05 | 0.00 | 0.27 | 0.00 | 2.55 | 0.13 | 0.01 | 4 | 31.3 |
| PE29 A | 0.07 | 0.12 | 0.00 | ND | 0.00 | ND | 0.14 | 0.02 | 2 | 37.5 |
| PPE47 A | ND | 0.17 | 0.01 | 0.03 | 0.01 | 0.00 | 0.15 | 0.01 | 2 | 43.8 |
| PPE47 B | 0.01 | 0.14 | 0.00 | 0.00 | 0.00 | 0.00 | 0.02 | ND | 1 | 12.5 |
| PPE47 C | 0.00 | 0.07 | 0.00 | 0.00 | 0.00 | 0.00 | 0.06 | 0.01 | 0 | 13.3 |
| PPE47 D | ND | 0.08 | 0.00 | 0.00 | ND | 0.00 | 0.04 | 0.02 | 0 | 13.3 |
| PE13 A | 0.20 | 0.19 | 0.03 | 0.18 | 0.03 | 0.80 | 0.15 | 0.03 | 5 | 82.4 |
| PPE46 A | ND | 0.16 | 0.00 | ND | 0.00 | 0.00 | 0.09 | 0.02 | 1 | 13.3 |
| PPE46 B | ND | 0.14 | 0.05 | 0.03 | 0.00 | 0.00 | 0.15 | ND | 2 | 40.0 |
| PPE18 A | 0.55 | 0.16 | 0.08 | 0.30 | 0.02 | 1.44 | 0.04 | 0.04 | 4 | 82.4 |
| PPE18 B | 0.19 | 0.06 | 0.09 | 0.15 | 0.02 | 0.44 | 0.07 | 0.01 | 3 | 88.2 |
| PPE18 C | 0.25 | 0.15 | 0.01 | 0.13 | 0.00 | 0.24 | 0.05 | 0.05 | 4 | 70.6 |
| PPE18 D | 0.23 | 0.17 | ND | 0.05 | 0.00 | 0.03 | 0.02 | 0.03 | 2 | 41.2 |
| PPE18 E | 0.17 | 0.15 | 0.00 | 0.01 | ND | 0.00 | 0.00 | 0.02 | 2 | 29.4 |
| PE8 A | 0.17 | 0.17 | 0.00 | ND | 0.01 | 0.14 | 0.02 | 0.04 | 3 | 31.3 |
| PE8 B | ND | 0.17 | 0.00 | 0.10 | 0.00 | 0.47 | 0.00 | ND | 2 | 29.4 |
| PE8 C | ND | 0.16 | 0.00 | 0.16 | 0.00 | 0.82 | 0.00 | 0.03 | 3 | 23.5 |
| PPE15 A | ND | 0.11 | ND | 0.41 | 0.00 | 1.19 | 0.00 | 0.01 | 3 | 31.3 |
| PPE15 B | 0.02 | 0.08 | 0.00 | ND | 0.02 | 0.00 | 0.00 | 0.00 | 0 | 11.8 |
| PPE15 C | ND | 0.04 | ND | ND | ND | 0.18 | 0.02 | 0.00 | 1 | 17.6 |
| PPE15 D | ND | 0.06 | 0.00 | ND | 0.01 | 0.00 | 0.00 | 0.01 | 0 | 6.3 |
| PPE15 E | 0.02 | 0.06 | 0.00 | ND | 0.01 | 0.02 | 0.00 | 0.00 | 0 | 6.3 |
| PE32 A | 0.03 | 0.10 | 0.00 | ND | 0.00 | 0.05 | 0.00 | ND | 1 | 17.6 |
| PPE65 A | ND | 0.10 | 0.01 | ND | 0.02 | 0.10 | 0.00 | 0.01 | 1 | 26.7 |
| PPE65 B | ND | 0.11 | 0.07 | ND | 0.02 | 0.00 | 0.00 | 0.01 | 1 | 50.0 |
| PPE65 C | 0.10 | 0.11 | 0.00 | 0.04 | 0.01 | 0.03 | 0.00 | 0.00 | 1 | 6.3 |
| PPE65 D | 0.05 | 0.07 | 0.00 | ND | 0.02 | 0.00 | ND | 0.00 | 0 | 5.9 |
| PPE65 E | 0.07 | 0.09 | 0.00 | 0.05 | 0.01 | 0.01 | ND | ND | 0 | 13.3 |
| PE22 A | 0.02 | 0.06 | 0.00 | 0.06 | ND | 0.02 | ND | 0.00 | 0 | 0.0 |
| PPE36 A | 0.10 | 0.10 | 0.00 | 0.21 | ND | 0.92 | ND | 0.02 | 2 | 23.5 |
| PPE36 B | 0.00 | 0.08 | 0.00 | 0.01 | ND | 0.00 | ND | 0.00 | 0 | 0.0 |
| PPE36 C | 0.00 | 0.10 | 0.00 | 0.01 | ND | 0.00 | 0.00 | 0.01 | 1 | 0.0 |
| PE25 A | 0.00 | 0.16 | ND | 0.00 | 0.00 | 0.00 | ND | 0.01 | 1 | 0.0 |
| PPE41 A | 0.09 | 0.06 | 0.00 | 0.08 | ND | 0.41 | ND | 0.00 | 1 | 28.6 |
| PPE41 B | 0.03 | 0.09 | 0.00 | 0.00 | 0.01 | 0.00 | 0.00 | 0.01 | 0 | 0.0 |
| PE11 A | 0.04 | 0.11 | 0.00 | 0.00 | 0.01 | 0.00 | 0.00 | 0.00 | 1 | 0.0 |
| PPE17 A | 0.01 | 0.09 | 0.00 | 0.05 | ND | 0.00 | 0.00 | ND | 0 | 12.5 |
| PPE17 B | 0.00 | 0.08 | 0.00 | ND | 0.01 | 0.00 | 0.00 | 0.01 | 0 | 0.0 |
| PPE17 C | 0.00 | 0.08 | 0.00 | 0.08 | 0.00 | 0.00 | 0.00 | ND | 0 | 0.0 |
| PPE17 D | ND | 0.14 | ND | ND | 0.00 | 0.05 | 0.00 | 0.00 | 1 | 7.1 |
| PE20 A | 0.12 | 0.04 | 0.00 | 0.17 | ND | 0.52 | 0.00 | 0.00 | 3 | 41.2 |
| PPE31 A | 0.21 | 0.10 | 0.01 | 0.27 | ND | 1.09 | 0.01 | 0.00 | 4 | 25.0 |
| PPE31 B | 0.12 | 0.04 | 0.02 | 0.11 | 0.02 | 0.55 | 0.01 | 0.00 | 3 | 41.2 |
| PPE31 C | 0.04 | 0.07 | 0.00 | 0.00 | 0.00 | 0.01 | 0.01 | 0.00 | 0 | 6.3 |
| PPE31 D | 0.02 | 0.05 | 0.00 | 0.01 | ND | 0.00 | 0.00 | 0.00 | 0 | 0.0 |
| PPE31 E | 0.04 | 0.01 | 0.00 | 0.00 | 0.00 | ND | 0.00 | ND | 0 | 0.0 |
| PE31 A | 0.25 | 0.04 | 0.01 | 0.23 | 0.00 | 1.05 | 0.01 | 0.00 | 3 | 64.7 |
| PPE60 A | 0.29 | 0.10 | 0.00 | 0.29 | 0.00 | 1.43 | 0.02 | ND | 3 | 68.8 |
| PPE60 B | 0.28 | 0.08 | 0.00 | 0.11 | 0.00 | 0.58 | 0.00 | 0.00 | 3 | 47.1 |
| PPE60 C | 0.25 | ND | 0.00 | 0.19 | 0.01 | 0.89 | 0.03 | 0.00 | 3 | 46.7 |
| PPE60 D | 0.52 | 0.06 | 0.01 | 0.18 | ND | 1.03 | 0.01 | 0.00 | 3 | 35.3 |
| PPE60 E | 0.40 | 0.11 | 0.00 | 0.15 | 0.00 | 1.54 | 0.01 | 0.00 | 4 | 23.5 |
| PE27 A | 0.23 | 0.06 | 0.00 | 0.22 | 0.00 | 0.83 | 0.00 | 0.00 | 3 | 25.0 |
| PE27 B | 0.25 | 0.05 | 0.01 | 0.13 | ND | 1.20 | 0.00 | 0.00 | 3 | 35.7 |
| PE27 C | 0.16 | 0.04 | 0.00 | 0.12 | 0.00 | 0.54 | 0.00 | 0.00 | 3 | 21.4 |
| PPE43 A | 0.30 | 0.12 | 0.00 | 0.23 | ND | 1.90 | 0.00 | 0.00 | 4 | 35.3 |
| PPE43 B | 0.00 | 0.13 | ND | 0.00 | ND | 0.00 | 0.01 | 0.00 | 1 | 14.3 |
| PPE43 C | 0.01 | 0.12 | 0.00 | 0.00 | 0.00 | 0.00 | 0.00 | 0.00 | 1 | 7.1 |
| PPE43 D | 0.04 | 0.10 | 0.01 | 0.01 | 0.00 | 0.00 | 0.03 | 0.02 | 1 | 6.3 |
| PPE43 E | 0.01 | 0.00 | 0.00 | 0.08 | 0.00 | 0.47 | 0.00 | 0.00 | 1 | 18.8 |
| PE7 A | 0.06 | 0.03 | 0.00 | 0.02 | 0.00 | 0.05 | 0.01 | 0.00 | 0 | 7.1 |
| PPE14 A | 0.28 | 0.06 | 0.00 | 0.27 | 0.00 | 1.48 | 0.00 | 0.00 | 3 | 26.7 |
| PPE14 B | ND | 0.04 | 0.01 | 0.13 | 0.00 | 0.16 | 0.01 | 0.00 | 2 | 6.3 |
| PPE14 C | 0.05 | 0.00 | 0.00 | 0.13 | 0.00 | 0.00 | 0.00 | 0.00 | 1 | 0.0 |
| PPE14 D | 0.10 | 0.15 | 0.00 | 0.12 | 0.00 | 0.31 | ND | 0.00 | 4 | 18.8 |
| PPE14 E | 0.02 | 0.04 | 0.00 | 0.00 | 0.00 | 0.00 | ND | 0.00 | 0 | 13.3 |
| PE15 A | 0.07 | 0.02 | 0.00 | 0.02 | ND | 0.07 | ND | 0.00 | 0 | 41.2 |
| PPE20 A | 0.12 | 0.01 | 0.04 | 0.09 | 0.00 | 0.20 | ND | 0.00 | 2 | 80.0 |
| PPE20 B | 0.06 | 0.15 | 0.02 | 0.04 | 0.00 | 0.05 | 0.02 | 0.00 | 1 | 66.7 |
| PPE20 C | 0.05 | 0.02 | 0.01 | 0.02 | 0.00 | 0.00 | 0.01 | 0.00 | 0 | 35.7 |
| PPE20 D | 0.00 | 0.03 | ND | 0.00 | ND | 0.00 | ND | 0.00 | 0 | 21.4 |
| PPE20 E | 0.01 | 0.00 | 0.00 | 0.00 | 0.05 | ND | ND | 0.00 | 0 | 7.7 |
| PPE20 F | 0.02 | 0.00 | ND | 0.00 | 0.00 | 0.00 | ND | 0.00 | 0 | 0.0 |
|  |  |  |  |  |  |  |  |  |  |  |
| **# pools recognised** | **29** | **40** | **0** | **27** | **0** | **34** | **8** | **0** | **37** |  |
